# Missed Essex-Lopresti Injury—Development of a Combined Proximal and Distal Radio-Ulnar Joint Prosthesis as a Treatment Option and Proof of Concept

**DOI:** 10.3390/healthcare11162274

**Published:** 2023-08-11

**Authors:** Simon Oeckenpöhler, Martin Franz Langer, Oliver Riesenbeck

**Affiliations:** Department of Trauma, Hand and Reconstructive Surgery, University Hospital Münster, Waldeyer Str. 1, 48149 Muenster, Germany; simon.oeckenpoehler@ukmuenster.de (S.O.);

**Keywords:** Essex-Lopresti, DRUJ prosthesis, PRUJ prosthesis, interosseous membrane, salvage procedure, radial head fracture, radial head resection, complication, one bone forearm

## Abstract

Essex-Lopresti injuries are characterized by injuries to the proximal radio-ulnar joint, the distal radio-ulnar joint, and the interosseous membrane. This can lead to osteoarthritis, impaction syndrome, or instability. If all three structures are injured and lead to instability, the situation is almost unmanageable and many times ends in a one-bone forearm. In this article, we demonstrate a new way to reconstruct the proximal and distal radio-ulnar joint with two patient-specific coupled prostheses. These have been developed with the biomechanical conditions of the forearm in mind, where there are very large forces between the bones. As a result, we are able to present a patient previously severely restricted in the use of his hand and arm via a splint that compressed the forearm, who is now able to perform everyday activities and even light sports, such as badminton, without pain.

## 1. Introduction

The triad of a radial head fracture, interosseous membrane rupture and instability, and distal radio-ulnar joint (DRUJ) instability was first published in two cases by Essex-Lopresti in 1951 [1]. Since then, various treatment algorithms have been developed to restore optimal function and stability to the forearm, but no optimal solution has been established. In particular, the treatment of chronic cases with high-grade, persistent instability often results in poor functional outcomes.

Wegmann et al. [2] used a high-speed camera to analyze the mechanism of the complete separation of the forearm bones. They found that after a fracture of the proximal radius, the interosseous membrane tears down to the distal ulna, and the wrist attachment apparatus becomes unstable.

Early recognition of the injury is crucial to allow proper healing and avoid costly revisions. Patients usually present with combined elbow and wrist pain. In cases of greater instability, the distal symptoms correspond to ulnar impingement with proximal radial migration. This can also occur during the course of the disease. In addition, there is often a general feeling of instability and associated functional limitations. All of these symptoms initially raise the suspicion of a high-grade lesion in radial head fractures and should be investigated further. Schnetzke et al. [3] described in 2017 that 48% of their patients only received a correct diagnosis after more than four weeks, and therefore, primary care was not possible.

Different treatment algorithms for acute injuries are described, whereby recent literature recommends reconstruction of the radial column (radial head osteosynthesis or replacement) along with stabilization of the distal radio-ulnar joint [4].

Long-term injuries usually lead to chronic symptoms and are difficult to treat, as Matthias and Wright pointed out in a review in 2016 [5], which Schnetzke et al. confirmed in 2017 with a study of 31 patients, half of whom were treated more than one month after injury [3]. Late presentation requires radial length reconstruction, which is only possible with prosthetic replacement, to prevent proximal migration [6,7,8]. If radial length reconstruction is not possible due to a lack of proximal resistance, prosthesis dislocation, etc., wrist pain can be anticipated as a result. In this situation, Jungbluth et al. recommended distal radio-ulnar fusion, which was described by Sauvé-Kapandji with acceptable results [9]. Others recommend reconstruction of the interosseus membrane; however, more cadaver studies than in vivo studies have been published on this topic [10,11,12,13,14,15] due to the rarity of the injury and the resulting small number of cases. However, reconstruction of the membrane only has a chance of success if the bony situation is stable or replaced, especially proximally, and a sufficiently stable retaining apparatus can also be constructed distally. 

Most reports of long-term failed treatment of an Essex-Lopresti injury result primarily in a dysfunctional arm with chronic pain, which is ultimately resolved by the creation of a one-bone forearm. However, in 1995 Peterson et al. reported on the complications of this salvage surgery [16]. With primary healing rates of 68%, secondary healing rates of 74%, and a functional outcome of 31% fair and poor results, this option is poorly predictable and also unsatisfactory.

Thus, there is a lack of good solutions for patients with forearm instability on the basis of an unrecognized and/or misdiagnosed and subsequently poorly treated Essex-Lopresti lesion with a bony defect. The following article presents an alternative therapeutic option to a one-bone forearm for this difficult-to-treat, chronic-stage injury. The development of a distal and proximal radio-ulnar joint prosthesis, taking into account the biomechanical requirements, is explained using a case example.

## 2. Materials and Methods

**Figure 1:**
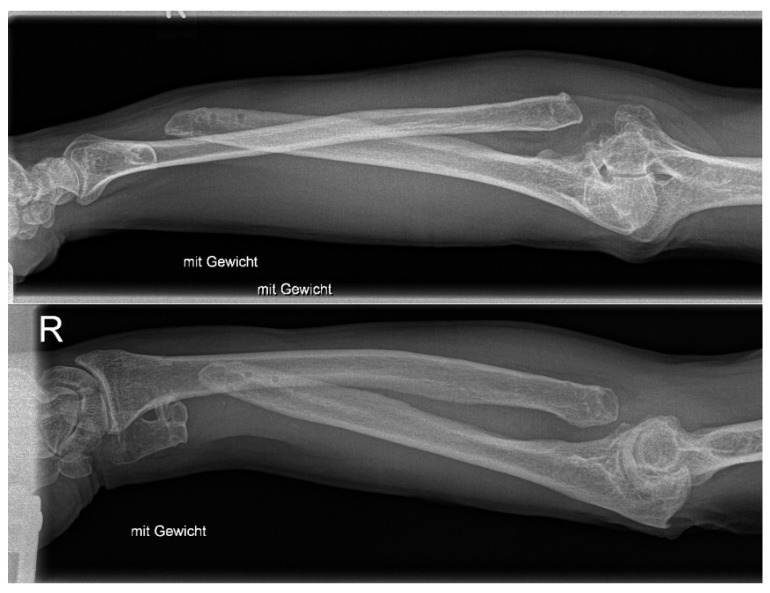
A.P. and axial X-ray of the unstable forearm under load after radial head resection, failed ulna shortening osteotomy, Sauvé-Kapandji procedure, and removal of all implants.

The patient we present had a motorcycle accident and underwent ex situ reconstruction of the radial head at the age of 41 with the subsequent need for a prosthesis. After the prosthesis loosened, it was removed, and an ulnar-shortening osteotomy was performed due to chronic wrist pain. Due to recurrent complaints, a Sauvé-Kapandji procedure was performed, followed by removal of the implant. The complex instability of the forearm was misunderstood throughout the course of care, up to the point when the patient presented to our clinic thirteen years after the injury. The gross instability is shown in the X-ray image of the forearm under load (Figure 1). The patient was unable to write with this hand prior to the injury dominant hand. Disabilities of Arm, Shoulder, and Hand Score was 79, grip strength on the Jamar hydraulic hand dynamometer (Performance Health Supply, Cedarburg, WI, USA) was 11 kg (44 kg on contralateral side), and pronation/supination was 10/0/10° (90/0/90° on contralateral side). The patient could only achieve a reduction in pain with low usability of the right arm through a tight cuff made of leather, which was tightly wrapped around the entire circumference of the arm and wrist every day. Without this cuff, the left hand had to support the right forearm in all positions to make movement possible. 

In order to find a therapeutic approach for this complex situation, we searched the literature, which unfortunately only recommends the creation of a one-bone forearm [16,17,18] to salvage this complex situation. 

As the function of a one-bone forearm is usually very unsatisfactory, we were looking for a solution to reconstruct the proximal radio-ulnar joint (PRUJ) with a destroyed capitulum and a reconstruction of the distal radio-ulnar joint with a stable lateral distance between the radius and the ulna to stabilize the forearm. The proximal radio-ulnar joint could only be attached to the ulna. Due to the destroyed capitulum and unstable ligamentous structures of the elbow joint, a common radial head prosthesis and capitulum replacement were not an option. In this case, the principle of the Scheker prosthesis [19,20,21] was optimal for the distal radio-ulnar joint, although a more stable version had to be made due to the grotesque instability. In the physiologically intact state, approx. 70% of the pressure from the hand is transferred to the radius, and approx. 30% to the ulna. Approximately 20% of this load is transferred from the radius to the ulna through the central part of the interosseous membrane. This results in a force transfer in the elbow area of approx. 50% (in supination) via the radius and 50% via the ulna [22]. In the case of grotesque instability, as described above, the load must be transferred via the two prosthetic components. This requires a significantly higher primary stability than possible with a standard prosthesis. After resection of the radial head, the force must also be completely transferred proximally to the ulnar component. For this purpose, we designed a rod that was anchored longways into the proximal ulnar shaft and into the distal radius in the opposite direction so that loosening of the prosthesis would be prevented. The two components were coated with hydroxyapatite to allow osteointegration of the construct and, thus, ensure the long life of the prosthesis. After planning, drawing, and optimizing several initial sketches, we found an industrial partner (Implantcast GmbH, Lüneburger Schanze 26D, 21614 Buxtehude, Germany) to assist in the planning and eventual manufacture of the prostheses. Normally, the cost of a custom-made prosthesis is 3–5 times that of a comparable standard prosthesis. However, such cases are so special that it depends on the individual case, and a cost between €10,000 and €25,000 can be expected.

We created a digital template using a 3D-reconstructed CT scan of the contralateral forearm, which was used to calculate the fitting. With this template, a necessary re-distalization of the radius by 10–15 mm was planned. This lengthening creates such high pressure from the soft tissues that support against the capitulum humeri is not sufficient in terms of direct pressure and protection against dislocation.

**Figure 2:**
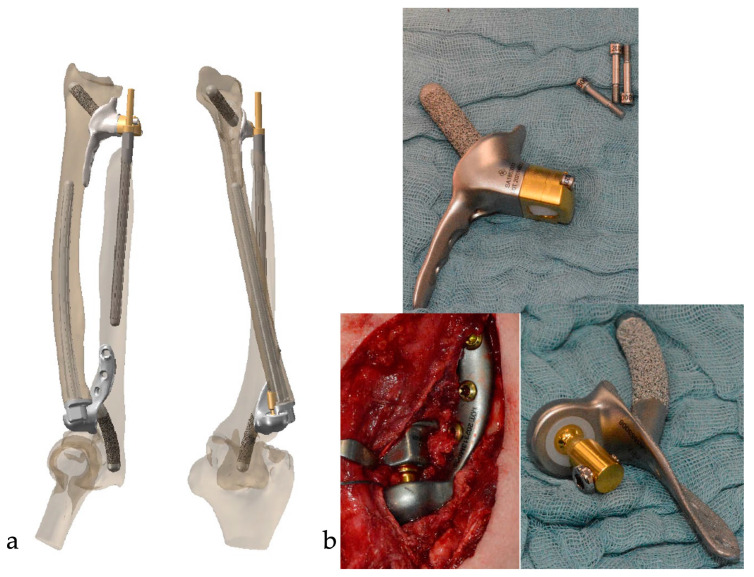
(**a**) 3D-planning of a patient-individualized combination of proximal and distal radio-ulnar joint replacement. (**b**) Original components and intraoperative picture of the proximal component in situ.

To provide the necessary stability proximally, the fixed part was screwed three times into the ulna, and more importantly, the above-mentioned rod was planned into the olecranon with a hydroxalappatatite-coated surface. The radial stem was planned to be 10 cm long, and since the resection and implantation levels could not be planned exactly preoperative, a telescopic mechanism was installed for fine adjustment. To avoid the problem of dislocation of the radial head prosthesis, we developed a semi-constrained prosthesis with a snap-fitting ball into the PE (see Figure 2). 

At the same time, a constrained distal ulna prosthesis was planned, which had to be more stable than the prostheses available on the market due to the high level of preoperative instability caused by the lack of the interosseous membrane. A total DRUJ replacement based on the development of Scheker et al. [19] was planned, which had a rod in the distal radius next to the triple screw connection, like the proximal radial part (see Figure 2), and a stem more than 7 cm long for the ulna.

Implantation of the proximal component required an unusual and untested approach. Therefore, the 3D jigged drill templates and plastic trial prostheses were delivered in multiple versions to find the best possible access to the proximal radio-ulnar joint and the best possible implantation technique with minimal risk to the adjacent neurovascular bundles.

## 3. Results

### Surgical Technique

Surgery was performed in one stage for both the proximal and distal replacement in the 55-year-old patient.

The patient was under general anesthesia because the estimated time of surgery was uncertain. He was placed in the supine position with his arm on a radiolucent side table. Prophylactic antibiotics were administered according to the in-house standard.

Due to multiple previous surgeries on both the proximal and distal forearm, the approaches were partially predetermined. 

The old dorsoradial scar at the elbow joint was reopened and dissected along the superior border of the anconeus muscle to the ulna. A short, sclerosed portion of the proximal radius was then resected to allow the component to be placed on the ulna. The first step was to prepare the hole for the rod in the proximal ulna. This was difficult in our case because of the hard bone and the curved rod. The radius was then prepared. It was reamed, and the joint component of the proximal radius could be inserted. The ulnar component was then inserted and screwed in place, and the two parts were connected. This step was very difficult due to high soft tissue tension. To allow for secondary lengthening, the proximal radius was gradually lengthened with distractors until the required length was achieved. After checking the fit and performing pro- and supination, and radiographic checks, the wound was closed layer by layer. Complete fascial closure was particularly important from our perspective.

Reconstruction of the distal radio-ulnar joint followed. A dorsal approach was made to the distal radio-ulnar joint. The extensor digiti minimi tendon was dissected from the 5th extensor compartment, and the bony area of the DRUJ arthrodesis was exposed. This area was completely resected. The triangular fibrocartilage complex (TFCC) was left intact to avoid opening the joint. The ulnar aspect of the distal radius was then exposed and smoothed. The drill guide was placed here, and the hole for the rod was drilled in the direction of the styloid process. The ulna was then opened distally with an awl and reamed to 7 mm. The ulnar component was then inserted into the shaft component. The distal radius component was then fixed to the ulnar side of the distal radius. The PE ball was then inserted into the new joint and tightened with the cover. Pro- and supination were checked. The extensor carpi ulnaris tendon was removed from the gliding channel, and the gliding channel was sutured over the prosthesis with 4-0 PDS. The capsule was also fixed with luxation of the extensor digiti minimi tendon. The wound was closed layer by layer.

The forearm was immobilized in a splint for the next four weeks. From the third post-operative day, the patient was allowed to perform pain-adapted exercises with physiotherapy, after which the splint was reapplied. Non-weight bearing was consistently maintained for eight weeks and then slowly increased.

**Figure 3:**
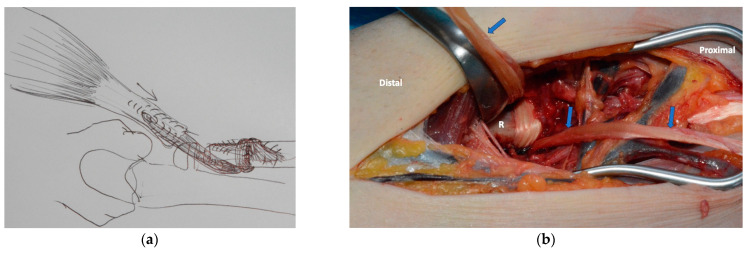
(**a**) Preoperative drawing of the biceps-reinsertion; (**b**) intraoperative picture with tendon graft in situ, R: Radius, Blue Arrows: Semi-T tendon.

During the previous surgical therapy, the attachment of the biceps tendon to the radial tuberosity was resected so that the patient was already unable to supinate the forearm preoperatively. During the initial surgical treatment, only the prosthesis was implanted. After the prosthesis had healed, the insertion of the biceps brachii muscle could be reconstructed in a further step. For this purpose, the retracted stump of the tendon had to be lengthened, which we performed utilizing the semitendinosus tendon. This was looped around the proximal radius and fixed to it. This type of tendon fixation resulted in long-distance contact of the tendon with the proximal radius adjacent to the implant and with the tendon itself, resulting in a stable situation during healing. At the same time, this created the largest possible lateral lever arm for supination in the absence of the radial tuberosity (see Figure 3).

**Figure 4:**
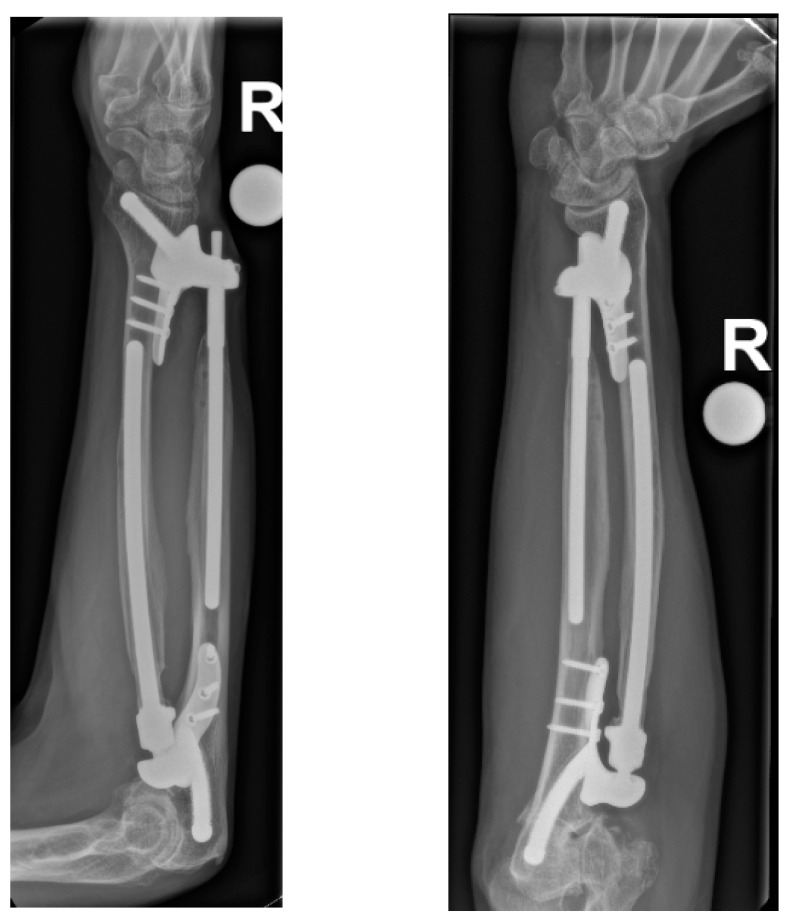
Follow-up X-ray at 2.5 years after combined proximal and distal DRUJ replacement.

This maneuver improved pronation/supination from 50/0/0° after prosthesis implantation to 90/0/45° at follow-up after a total of two and a half years (initially, it was 10/0/10° before presentation to our department). The mobility in the elbow is currently extension/flexion at 0/0/120°. The patient needed much physiotherapy and training to regain function and finally supinate the arm powerfully due to the retraction of the tendon and the long period of non-use. The DASH score improved from 79 to 42, and grip strength improved from 11 kg to 24 kg two and a half years after implantation. The patient started using the hand again for writing and holding a pen six months after prosthesis implantation. He has returned to playing badminton, which he now does without pain. The mobility of the wrist is currently extension/flexion 60/0/60° and radialduction/ulnarduction 20/0/25°. Finger extension and fist closure are still fully possible. The combined proximal and distal radio-ulnar prosthesis has now been in the forearm for two and a half years. The radiographs show that the prosthesis has healed, and there are no signs of secondary complications, such as stress shielding, loosening, etc. (Figure 4). The patient has almost regained his original life (“I have my old life back”). Of course, we and the patient are still cautious due to the lack of experience, as the options for a revision would open up a whole new chapter.

## 4. Discussion

To the best of our knowledge, the technique of single-stage surgical replacement of the proximal and distal radio-ulnar joint described in this paper is a new technique. It was used to stabilize a grotesque forearm instability after an initial Essex-Lopresti injury and to restore function to the patient’s arm. The follow-up at two and a half years is considered good for this type of treatment without secondary complications but requires further monitoring.

The proximal radio-ulnar joint (PRUJ) is a region where there are few and mostly unsatisfactory solutions for failed fractures of the radial head or capitulum, poor results with radial head prostheses, and disorders of pro- and supination. There is only one patent in the literature for the proximal radio-ulnar joint, by Louis Scheker in 2005, with a constrained replacement of the radial head with a prosthesis. The prosthesis was attached to the ulna with three screws and consisted of a spherical radial head prosthesis fixed in this radial head component [23]. We are not aware of any clinical cases with this prosthesis. The new way of reconstructing the PRUJ described here may open new doors, and this case has encouraged us to provide other similar cases with an individualized PRUJ prosthesis.

In another patient, we have already modified the prosthesis design to include a biceps insertion site on the proximal radius. We opted for a primary coated area with holes for tendon reinsertion but cannot report on the results yet.

The procedures mentioned in the introduction with a reconstruction of the membrane in the early stages are certainly preferable to the method presented here, although the results are very heterogeneous [13]. In our own experience with interosseous membrane reconstruction, the impaction syndrome of the proximal and distal radio-ulnar joint can be reduced by a very stable reconstruction of the interosseous membrane, but with very significant limitations on pro- and supination. Therefore, we no longer perform this procedure.

The success of this prosthesis–compared to other failed attempts to reconstruct the proximal radio-ulnar joint–can be seen in its great stability. This was achieved by the long intramedullary components in both proximal and distal bones. Although this increased the difficulty of the operation and the operating time, it was important in our opinion. According to unpublished conference reports, attempts to use a reverse Scheker prosthesis for the proximal radio-ulnar joint were unsuccessful or had little success. Attempts to fix this component with screws alone have also been unsuccessful. The patient-specific fabrication of the proximal component also allowed the exact location of the center of rotation of the original radial head to be determined, and the prosthesis was designed accordingly. In our opinion, this is an important factor because otherwise, the force vectors of the forearm muscles would lead to lateral deflections of the PRUJ prosthesis, which would result in loosening. 

Since the length ratio of the radius to the ulna changes only distally due to forearm rotation, there is no need for proximal length compensation. Therefore, the proximal component with a simple spherical joint–in the active position–is sufficient.

In this case, with a destroyed capitulum and a long-ago performed radial head resection, radial support in the former capitulum area would have been desirable but would have meant an additional prosthesis for the capitulum and the humero-radial joint. 

However, a problem remains in the proximal radio-ulnar joint regarding the attachment of the biceps tendon. The lateral pull of the biceps tendon on the radius when the elbow is flexed creates significant forces on the proximal radius, which could lead to increased deterioration or dislocation. Of course, we see the potential for further development in the direction of the patient’s supination, which is currently missing by about 30°. To optimize this, we have planned to surgically tighten the tendon.

On the other hand, for poorly or unfavorably treated cases, such as the one presented here, the creation of a one-bone forearm is, in our opinion, the only alternative. The creation of a one-bone forearm is cheaper, more valid, and more frequently tested but offers a much worse functional result than we were able to achieve in the patient described above.

## 5. Conclusions

The alternative treatment option presented here for an aggravated Essex-Lopresti injury is a completely new method for stabilizing a forearm. The injury and its consequences are fortunately rare, but this method may become an option for better function as an alternative to the one-bone-forearm. Further validation in large patient cohorts will be needed in the future to fully assess the value of this approach. 

## Data Availability

All existing data are presented in the article.
